# Inhibition of Parkinsonian tremor with cutaneous afferent evoked by transcutaneous electrical nerve stimulation

**DOI:** 10.1186/s12984-017-0286-2

**Published:** 2017-07-14

**Authors:** Man-Zhao Hao, Shao-Qin Xu, Zi-Xiang Hu, Fu-Liang Xu, Chuan-Xin M. Niu, Qin Xiao, Ning Lan

**Affiliations:** 1 Laboratory of Neurorehabilitation Engineering, School of Biomedical Engineering, Shanghai Jiao Tong University, 1954 Hua Shan Road, Shanghai, 200030 China; 2 Department of Neurology, School of Medicine, Shanghai Jiao Tong University, Shanghai, China; 3 Department of Rehabilitation, School of Medicine, Shanghai Jiao Tong University, Shanghai, China; 4 Division of Biokinesiology and Physical Therapy, Herman Ostrow School of Dentistry, University of Southern California, Los Angeles, USA

**Keywords:** Parkinson’s disease, Resting tremor, Spinal interneurons, Cutaneous reflexes, Transcutaneous electrical nerve stimulation

## Abstract

**Background:**

Recent study suggests that tremor signals are transmitted by way of multi-synaptic corticospinal pathway. Neurophysiological studies have also demonstrated that cutaneous afferents exert potent inhibition to descending motor commands by way of spinal interneurons. We hypothesize in this study that cutaneous afferents could also affect the transmission of tremor signals, thus, inhibit tremor in patients with PD.

**Methods:**

We tested this hypothesis by activating cutaneous afferents in the dorsal hand skin innervated by superficial radial nerve using transcutaneous electrical nerve stimulation (TENS). Eight patients with PD having tremor dominant symptom were recruited to participate in this study using a consistent experimental protocol for tremor inhibition. Resting tremor and electromyogram (EMG) of muscles in the upper extremity of these subjects with PD were recorded, while surface stimulation was applied to the dorsal skin of the hand. Fifteen seconds of data were recorded for 5 s prior to, during and post stimulation. Power spectrum densities (PSDs) of tremor and EMG signals were computed for each data segment. The peak values of PSDs in three data segments were compared to detect evidence of tremor inhibition.

**Results:**

At stimulation intensity from 1.5 to 1.75 times of radiating sensation threshold, apparent suppressions of tremor at wrist, forearm and upper arm and in the EMGs were observed immediately at the onset of stimulation. After termination of stimulation, tremor and rhythmic EMG bursts reemerged gradually. Statistical analysis of peak spectral amplitudes showed a significant difference in joint tremors and EMGs during and prior to stimulation in all 8 subjects with PD. The average percentage of suppression was 61.56% in tremor across all joints of all subjects, and 47.97% in EMG of all muscles. The suppression appeared to occur mainly in distal joints and muscles. There was a slight, but inconsistent effect on tremor frequency in the 8 patients with PD tested.

**Conclusions:**

Our results provide direct evidence that tremor in the upper extremity of patients with PD can be inhibited to a large extent with evoked cutaneous reflexes via surface stimulation of the dorsal hand skin area innervated by the superficial radial nerve.

## Background

About 70% of patients with Parkinson’s Disease (PD) exhibit resting tremor symptom [1, 2]. Parkinsonian resting tremor is an involuntary rhythmic movement mostly exhibited in hands and distal joints of the arms, but also occurring in head, leg and even trunk in some subjects with PD. Parkinsonian tremor in mild degree may not cause a disability, but it may present a problem in performing personal activities of daily living with trembling hands, such as ‘Eating’, ‘Eat, drink and cutting food’, ‘Walking in neighborhood’, and ‘Writing’ [3].

Treatments of PD tremor focus on medication [4], kinematic adjustment and compensation using functional electrical stimulation [5–7], and inhibition based on spinal stretch reflex mechanism via Ib inhibition by low transcutaneous electrical nerve stimulation [8]. But the underlying neural mechanisms or the effectiveness of current interventions need further clarification. Tremor recalcitrant to oral medication remains a criterion to recommend patients with PD for deep brain stimulation (DBS) surgery. DBS has been an effective treatment for PD symptoms including tremor, but it is often recommended for severe cases [9–11].

It is well established that Parkinsonian tremor is originated from malfunctioning cerebral networks [12–15]. Two central oscillations at tremor and double tremor frequencies are identified in subjects with PD. It is found that the cortical signal at double tremor frequency dominates the cerebral activity and provides a direct drive to peripheral muscle activities [15], while the cortical signal at single tremor frequency acts primarily to synchronize activities of a group of peripheral muscles in modulating tremor intensity [16]. Timmermann et al. [17] suggested a flip-flop switch in the spinal cord that can direct the cortical oscillation signal at double tremor frequency to flexor and extensor muscles, so that an alternating bursting pattern of muscle activation is created.

There is a large body of neurophysiological evidence in cats and macaque monkeys that revealed that propriospinal neurons (PNs) in the C3-4 spinal cord mediate voluntary commands from the motor cortex and project directly to forelimb motor neurons (MNs) [18–23]. Based on the evidence in animals and human, Hao et al. [24] developed a computational model of PNs, and proposed a hypothetical model that may explain the role that the PN plays in computing the alternating rhythmic activation of a pair of antagonist muscles from cortical oscillations of single and double tremor frequencies in patients with PD. These studies indicate a potential spinal neural mechanism for interfering tremor signal transmission, particularly, PNs interact with a rich variety of afferents, including cutaneous afferent [25].

Direct evidence showed that cutaneous reflex evoked by stimulating finger skin typically exhibits a tri-phasic compound response in muscle EMG of both normal and subjects with PD [26, 27]. These responses include an early excitation of the motor neuron (MN), a 2nd large suppression of the MN, and a 3rd long latency excitation. The early excitation to MN is mediated by monosynaptic excitation; the 2nd suppression is via a pre-MN interneuron with a slightly longer latency; and the 3rd excitation matches to the long time delay of the transcortical loop response. Electrical stimulation of the pure cutaneous superficial radial nerve of the ipsilateral forelimb in cat evoked strong disynaptic inhibitory postsynaptic potentials (IPSPs) [25], which were presumably via the PN. The inhibitory effect of cutaneous reflex via pre-MN interneurons observed in both animals and human suggests a potential neural pathway to affect cortical tremor signals.

Taking together all evidence in previous studies, we hypothesize in this study that tremor intensity in patients with PD could be reduced by evoked cutaneous afferents with stimulation of the superficial radial nerve. The objective here was to test this hypothesis in subjects with PD and to provide direct evidence of tremor inhibition by cutaneous reflexes evoked with transcutaneous electrical nerve stimulation (TENS). We performed preliminary tests in subjects with PD to demonstrate the feasibility that cutaneous stimulation could produce an inhibitory effect on tremor. Immediate inhibition of joint tremors and EMGs of muscles during cutaneous stimulation was observed [28]. The preliminary tests confirmed the feasibility of tremor inhibition by evoked cutaneous reflexes. In this study, a rigorous paradigm was designed to evoke cutaneous reflex and to quantify the inhibitory effects of cutaneous reflex in both tremor amplitudes and EMGs of muscles in the upper extremity in another 8 subjects with PD. Results obtained in this study supported the above hypothesis and provided direct evidence that cutaneous afferents evoked by stimulation peripheral skin receptors innerved by the superficial radial nerve can diminish tremor intensity in subjects with PD.

## Methods

### Subjects

Eight idiopathic Parkinson’s disease patients with tremor dominant symptoms were recruited from the Department of Neurology, Ruijin Hospital for this study. The subjects were evaluated for their relative intensiveness of tremor symptom using the UPDRS scale. All subjects were asked to take their morning medication as usual before 7:00 am on the day of test. Experiments were performed between 10:00 am and 11:30 am before the noon medication was given. The information of the recruited subjects with Parkinson’s disease is presented in Table 1. The Ethics Committee of Animal and Human Subject Studies of Med-X Research Institute, Shanghai Jiao Tong University, approved this study. The subjects were given the consent form before joining the study.

**Table 1:** Subject information

Subjects	Gender	Test side^a^	Age (*yrs.*)	Disease course (*yrs.*)	UPDRS part III^b^	UPDRS Resting tremor score	L-Dopa Equivalents (mg/d)
PD1	F	R	60	6	16	2	300
PD2	F	R	62	5	22	3	100
PD3	F	L	56	1/2	15	2	0
PD4	M	R	58	5	17	2	375
PD5	M	R	67	9	18	2	450
PD6	M	L	76	7	23	2	525
PD7	F	R	62	2	17	2	75
PD8	M	L	69	3	20	2	525

^a^Test Side was chosen by tremor originated side of PD subject

^b^UPDRS Part III stands for Motor Section III (0 ~ 56) of the Unified Parkinson’s Disease Rating Scale. Tremor severity was evaluated using item 20 of UPDRS (Resting Tremor Score)

### Experimental protocol

Experimental protocol was developed in a preliminary study [28]. The procedures described below were adopted in experiments for quantifying tremor inhibition in this study.

#### Experiment setup and data acquisition

**Fig. 1:**
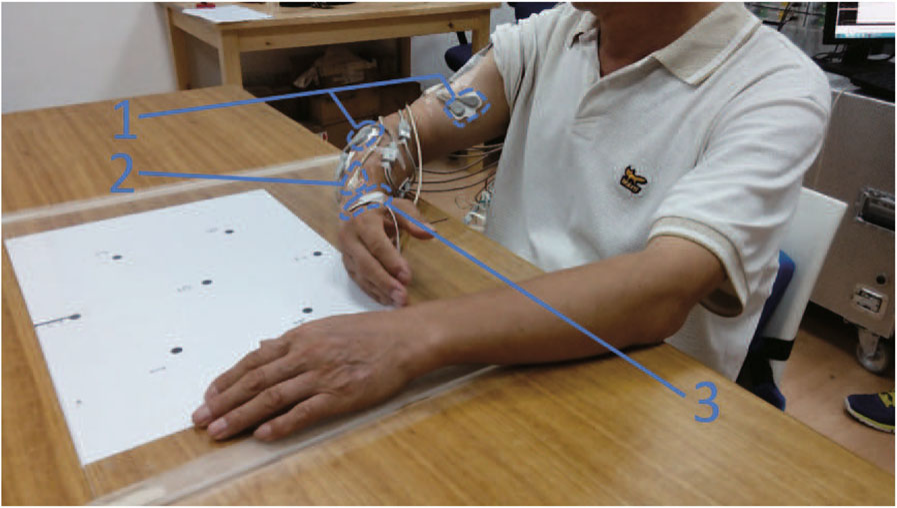
Experimental setup. Subjects sit comfortably with their arms supported by a desk. The Electromyography (EMG) signal of a muscle was recorded with bipolar surface electrodes (label 1). EMGs and kinematic data were simultaneously recorded from one tremor-affected arm. Six muscles were: biceps brachii (Biceps), triceps brachii (Triceps), flexor carpi ulnaris (FCU), extensor carpi radialis (ECR), flexor digitorum superficialis (FDS) and extensor digitorum (ED). Arm movement was collected by the motion sensor (label 2) in a magnetic field. Seven degrees of freedom (DOFs) in joints were: wrist flexion/extension (Wr_F), wrist radial/ulnar flexion (Wr_R), forearm pronation/supination (FA_P), elbow flexion/extension (El_F), shoulder flexion/extension (Sh_F), shoulder rotation (Sh_R) and shoulder adduction/abduction (Sh_A). The electrodes of electrical stimulation (label 3) were placed on the dorsal skin of the hand along the distribution of the superficial radial nerve

In this experiment, the subject was seated in a chair in front of a table with adjustable height, and the subject’s forearm rested comfortably on the surface of the table (Fig. 1). In this way, the arm of the PD subject did not have to maintain a posture, thus, generating a resting tremor.

Surface electromyography (EMG) activity was recorded from the biceps brachii (Biceps), the triceps brachii (Triceps), the flexor carpi ulnaris (FCU), the extensor carpi radialis (ECR), the Flexor digitorum superficialis (FDS) and the extensor digitorum (ED) muscles. Silver/silver chloride (Ag/AgCl) bipolar electrodes (Norotrode™ Model BS-24SAF) were placed onto the belly of the targeted muscle along the muscle fiber direction and a copper reference electrode was placed in the lower back of the subject to minimize the effect of motion on EMG recording. The EMG signals were pre-amplified by 5000 times, with a low-pass (1 *Hz*, 4th-order) filter and a high-pass (1000 *Hz*, 2nd-order) filter set at the Grass™ LP511 AC amplifiers (Grass Technologies, Astro-Med, Inc.). The raw EMGs were then sampled at a rate of 2410 *Hz* by the A/D convertor of the MotionMonitor II system. Kinematics data of the upper limb were recorded using the MotionMonitor II System (The Innovative Sports Training, Inc. Chicago, IL, USA) at a sampling rate of 120 *Hz*, and then linearly interpolated to align to the EMG sampling rate at 2410 *Hz* for synchronized timing of data points. Seven degrees of freedom (DOFs) of upper extremity trembling motion were examined in this study, i.e. the shoulder flexion/extension (Sh_F), the humeral rotation (Sh_R), the shoulder adduction/abduction (Sh_A), the elbow flexion/extension (El_F), the forearm pronation/supination (FA_P), the wrist flexion/extension (Wr_F) and the wrist radial/ulnar flexion (Wr_R).

#### Cutaneous stimulation

Cutaneous afferents with perception were evoked by surface electrical stimulation using a bipolar non-woven surface electrode (25 mm diameter, CM25RC, Cathay Manufacturing Crop. Shanghai, China). The electrode was placed on the dorsal skin of the hand near the metacarpophalangeal joint of index finger (covering the first interosseous space) as shown in Fig. 1. This part of skin was within the innervation zone of the superficial radial nerve, and was the site normally employed to evoke cutaneous reflexes in neurophysiological studies [29–31].

We used a programmable stimulator (Master-9, A.M.P.I Inc. Israel) to generate a train of biphasic, charge balanced current pulses with 200 μs pulse width at 250 *Hz* pulse frequency. The pulse amplitude of stimuli was adjusted during the experiment to examine the effect of stimulation strength on tremor inhibition.

#### Identification of sensory threshold

After the electrodes were placed, electrical stimulation was delivered in incremental steps. The amplitude of stimuli was initially set at 1.5 *mA* and gradually increased at an interval of 0.25 *mA* until the subject felt a noticeable sensation at the dorsal skin. The stimulus amplitude at this level was called the cutaneous perceptual threshold. We then continued to increase the stimulus amplitude until the subject perceived a radiating sensation that ran from the dorsal skin to the fingers. The stimulus amplitude at this level was called the radiating threshold (RT) that produced a clear radiating paresthesia in the subjects [30]. The RT was used as a sensory marker in neurophysiological studies because this indicated that the superficial radial nerve beneath the skin was actually activated by surface electrical stimulation [21–23]. Stimulus intensity above the RT had been used to evoke cutaneous reflex in neurophysiological studies [32].

Once the stimulus intensity at the RT was identified, trials were conducted in some patients initially at and above the RT. The stimulus intensity at the values from 1.5 to 1.75 times RT was found to produce significant inhibition in the EMGs and DOFs and was used for all trials in all patients.

#### Experimental trials

After above steps, subjects were asked to relax, then put their forearm on the table without having to maintain a posture. During the experiment, they were instructed to look forward and counted numbers backwards from 100 to 2 clearly to divert their attention away from the resting tremor. This was reported as an effective way to stabilize the tremor in patients with PD [33]. In these trials, we chose not to cover the eyes of the subjects, since covering their eyes may aggravate their resting tremor. An epoch of data in each experimental trial was recorded, consisting of 5 *s* of tremor recording without stimulation (prior to stimulation), 5 *s* of cutaneous stimulation in the middle (during stimulation), and 5 *s* post stimulation (after stimulation). Sufficient time before recording in each trial was allowed for the tremor to occur and to stabilize. The amplitude of stimulation current was varied from 1.5 to 1.75 times of RT until inhibition of tremor occurred. The number of trials depended on the progress of experiments with each subject in 1.5 h’ duration. The total number of trials (n) for each subject, thus, varied from 9 to 13 as listed in Table 2.

**Table 2:** The total number of trials and the number of trials with augmentation in DOFs and EMGs

	n	EMGs						DOFs						
		FDS	ED	FCU	ECR	Biceps	Triceps	Wr_F	Wr_R	FA_P	El_F	Sh_F	Sh_R	Sh_A
PD1	9	3	2	3	2	2	2	0	1	1	3	3	1	1
PD2	12	2	1	1	0	3	1	1	2	1	3	4	1	3
PD3	11	0	0	0	0	1	0	0	1	0	0	2	2	1
PD4	11	0	0	2	0	3	5	0	0	0	0	0	1	4
PD5	9	1	0	1	0	1	2	4	0	0	0	2	2	2
PD6	9	2	0	3	0	2	0	0	0	0	0	1	2	3
PD7	13	0	1	1	0	2	4	0	1	5	5	4	3	3
PD8	13	0	1	2	0	3	4	2	0	0	0	4	0	1

“n” denotes the total number of trials conducted in each subject

### Signal processing and data analysis

Six channels of EMG signals and kinematic data of 7 DOFs in the upper arm joints were analyzed off-line using MATLAB (Version: R2010a, MathWorks Inc.).

#### EMG and kinematic signal pre-processing

For raw EMG signals, notch filters of zero phase shift with a width of 1 *Hz* and 14th order Butterworth type were used first to eliminate noises from power lines at the center frequency of 50 *Hz* and higher harmonics up to 500 *Hz*. Since the frequency of electrical stimulation was at 250 *Hz*, any artifacts of electrical stimulation on EMGs would be eliminated by the notch filter at 250 *Hz*. Another source of noise was from the magnetic transmitter of the MotionMonitor II at 120 *Hz*. These noises were eliminated using the same notch filters at the center frequency 120 *Hz* and higher harmonics, up to 360 *Hz*. After notch filtering, a bidirectional 4-th order Butterworth IIR (infinite impulse response) filter with band-pass frequencies from 20 *Hz* to 390 *Hz* was employed to separate the EMG signals from low-frequency motion artifacts and high-frequency noise. Subsequently the filtered EMG signals were rectified, and low-pass filtered with a cut-off frequency at 50 Hz (4-th order Butterworth) for further analysis. The raw kinematic data of 7 DOFs in joints were low-pass filtered by a 16-th order Butterworth filter with 20 *Hz* cut-off frequency to remove high-frequency noise.

#### Estimation of tremor amplitude and frequency

Power spectral densities (PSDs) of kinematic data and EMG signals were computed using Welch’s method [34], which is available in MATLAB function (pwelch). In the 15 *s* of data of each trial, the PSDs were evaluated for the first segment of data (5 *s*) prior to stimulation, second segment of data (5 *s*) during stimulation and third segment of data (5 *s*) after stimulation. In calculating the PSD, we used the Hamming window with a window width of 2 *s* (or 4820 points) and a window overlap of 1 *s* (or 2410 points). The peak values in the PSD of EMGs and DOFs between 2 to 7 *Hz* were determined as the marker for tremor intensity. For the three segments of data in each trial, the tremor intensity prior to stimulation was denoted as P_*p*_, during stimulation as P_*s*_ and after stimulation as P_*a*_. The frequency corresponding to the peak value was determined as the tremor frequency, F_*p*_ (prior to stimulation), F_*s*_ (during stimulation) and F_*a*_ (after stimulation).

#### Calculation of the percentage of reduction

In examining the data during tremor inhibition, we noticed that some EMGs and DOFs in joints in a few trials displayed an augmentation during stimulation (P_*p*_ < P_*s*_). The number of trials with augmentation in either EMGs or DOFs were counted, and presented in Table 2.

To quantify the extent of tremor inhibition by electrical cutaneous stimulation, we define the percentage of reduction (*PR*) in tremor intensity in EMGs and DOFs as follows:

1 $$ PR={\frac{\sum_1^n{P}_p-\sum_1^n{P}_s}{\sum_1^n{P}_p}}^{\mathrm{x}}100\% $$

where n denotes the total number of trials performed in one subject.

The *PR* across all subjects was averaged from the *PR* data of 8 subjects of each EMG and DOF. The averaged *PR* across all subjects for EMGs was averaged from the *PR* data from the 6 EMGs. The averaged *PR* across all subjects for DOFs was averaged from the *PR* data from the 7 DOFs.

### Statistical analysis

To evaluate the effects of cutaneous reflex inhibition on tremor intensity, we tested the alternative hypothesis that the PSD amplitudes prior to stimulation were greater than that during stimulation. We chose the non-parametric Wilcoxon signed-rank test (W-test) because the sample size in each subject was relative small, and there was no valid assumption for a Gaussian distribution in the sample population [35]. The W-test was performed with tremor intensity data P_*p*_, P_*s*_ and P_*a*_ for 6 EMGs and 7 DOFs in joints for each subject. The alternative hypotheses: P_*p*_ is greater than P_*s*_, P_*p*_ is not equal to P_*a*_, and P_*s*_ is not equal to P_*a*_. The level of significance was set at *p* < 0.05. Tremor frequency is uniform across all EMGs and DOFs in a subject with PD [16]. Thus, tremor frequency of a subject was evaluated as the average frequency at the peak of the PSDs of EMGs and DOFs. The W-test was performed with tremor frequency data averaged F_*p*_, averaged F_*s*_ and averaged F_*a*_ to show the effects of cutaneous stimulation on tremor frequency. The level of significance was set at *p* < 0.05. Statistical tests were carried out with the R software [36].

## Results

### Effects of stimulation intensity

**Fig. 2:**
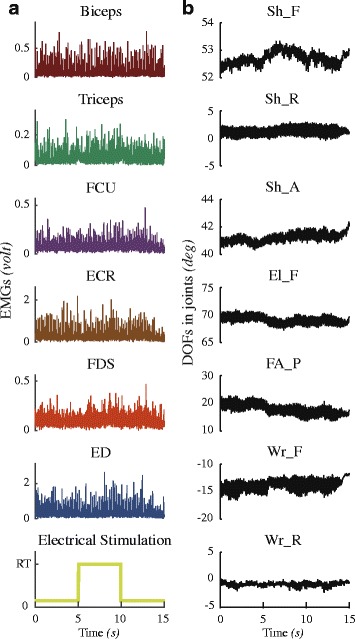
Results of one trial with low intensity stimulation amplitude of one subject (PD4). The amplitude of current stimulation was set at radiating threshold (RT). Column **a** shows the 6 preamplified EMGs during the trial. The seventh row shows the electrical stimulation. The time duration of one trial includes 5 *s* prior to stimulation, 5 *s* during stimulation and 5 *s* after stimulation. Column **b** shows 7 degrees of freedom (DOFs) in joints. During stimulation period, neither EMGs nor DOFs show an apparent reduction when electrical stimulation at RT

**Fig. 3:**
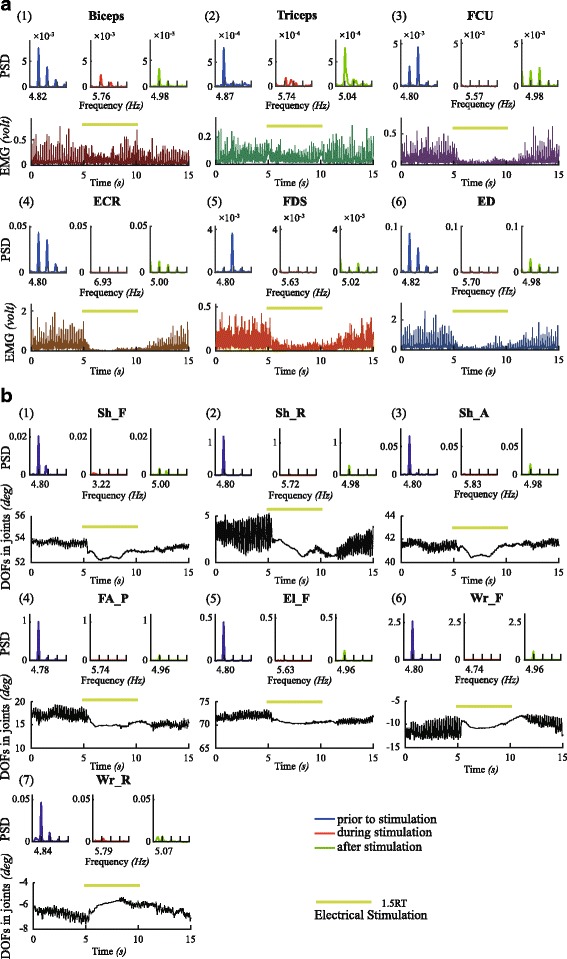
Results of high intensity stimulation amplitude at 1.5*RT in one subject (PD4). The figure shows the time series and the power spectral density (PSD) estimated by Welch method in three time periods during one trial of each EMG (**a**) and DOF (**b**). The horizontal line in chartreuse indicates the electrical stimulation in time series. PSDs of prior to stimulation are presented in blue, during stimulation in red and after stimulation in green. The frequency of the peak between 2 to 7 Hz in the PSD that calculated as the tremor frequency is listed

Stimulation intensity had significant impact on the effectiveness of cutaneous reflex inhibition to tremor. In each subject, we identified a stimulus threshold that produced a radiating sensation as reported in other studies [30, 31]. The values of the radiating threshold (RT) for each subject are listed in Table 3. We also tested some subjects using stimulation intensities below, at and above the radiating threshold. Typical examples of different stimulation intensities in one subject (PD4) are shown in Figs. 2 and 3. Figure 2 presents a trial with stimulation intensity set at the radiating threshold (RT). Inhibition to tremor activities was not apparent in the EMGs and joint DOFs. We also examined the power spectrum densities (PSDs) for these EMG and DOF data, no significant decrease in peak amplitudes during stimulation was observable. Only in ECR, there was a slight decrease in the PSD amplitude during stimulation.

**Table 3:** Radiating thresholds and stimulation amplitude

	Radiating threshold (RT) (*mA*)	Stimulation amplitude (RT)
PD1	5	1.75
PD2	5	1.5
PD3	5.5	1.5
PD4	4.5	1.5
PD5	3	1.5
PD6	2.5	1.5
PD7	3	1.5
PD8	4.5	1.5

However, when the stimulation intensity was increased to 1.5*RT, apparent reductions in the EMGs of 6 muscles and the tremor of the 7 DOFs in the shoulder, elbow and wrist were clearly observed in the time domain (Fig. 3). In the frequency domain, the peak values of the PSD of all DOFs at tremor frequency were reduced almost to zero (complete inhibition). A distinct decrease was also apparent in the peak amplitude of PSD for all EMGs. In the tests of all subjects, we found that if the stimulation intensity was set above the RT value from 1.5 to 1.75 times RT, significant inhibition in the EMGs and tremor at all DOFs was obtained. The values of stimulation amplitudes in each subject are also listed in Table 3.

### Effectiveness of inhibition

To quantify the effectiveness of cutaneous stimulation for tremor inhibition, the PSD of each trial prior to stimulation, during stimulation and after stimulation was calculated for each subject, as shown in Fig. 3 for subject PD4. The peak value of the PSD was identified in the frequency range between 2 to 7 *Hz*, and the frequency at the peak value was defined as the tremor frequency. Wilcoxon signed-rank test was performed to detect the significance in the difference between the PSD peaks (Table 4).

**Table 4:** Wilcoxon signed-rank test: prior to stimulation and during stimulation

	Biceps	Triceps	FCU	ECR	FDS	ED	Wr_F	Wr_R	FA_P	El_F	Sh_F	Sh_R	Sh_A
PD1	W = 32, Z = 1.13, *p* = 0.150, *r* = 0.27	W = 31, Z = 1.01, *p* = 0.180, *r* = 0.24	W = 28, Z = 0.65, *p* = 0.285, *r* = 0.15	W = 39, Z = 1.95, *p* = 0.027, *r* = 0.46	W = 26, Z = 0.41, *p* = 0.367, *r* = 0.10	W = 41, Z = 2.19, *p* = 0.014, *r* = 0.52	W = 45, Z = 2.67, *p* = 0.002, *r* = 0.63	W = 37, Z = 1.72, *p* = 0.049, *r* = 0.40	W = 41, Z = 2.19, *p* = 0.014, *r* = 0.52	W = 28, Z = 0.65, *p* = 0.285, *r* = 0.15	W = 31, Z = 1.01, *p* = 0.180, *r* = 0.24	W = 36, Z = 1.60, *p* = 0.064, *r* = 0.38	W = 38, Z = 1.84, *p* = 0.037, *r* = 0.43
PD2	W = 63, Z = 1.88, *p* = 0.032, *r* = 0.38	W = 77, Z = 2.98, *p* = 0.000~, *r* = 0.61	W = 71, Z = 2.51, *p* = 0.005, *r* = 0.51	W = 78, Z = 3.06, *p* = 0.000~, *r* = 0.62	W = 73, Z = 2.67, *p* = 0.002, *r* = 0.54	W = 76, Z = 2.90, *p* = 0.001, *r* = 0.59	W = 75, Z = 2.82, *p* = 0.001, *r* = 0.58	W = 74, Z = 2.75, *p* = 0.002, *r* = 0.56	W = 72, Z = 2.59, *p* = 0.003, *r* = 0.53	W = 63, Z = 1.88, *p* = 0.032, *r* = 0.38	W = 61, Z = 1.73, *p* = 0.046, *r* = 0.35	W = 77, Z = 2.98, *p* = 0.000~, *r* = 0.61	W = 65, Z = 2.04, *p* = 0.021, *r* = 0.42
PD3	W = 65, Z = 2.85, *p* = 0.001, *r* = 0.61	W = 66, Z = 2.93, *p* = 0.000~, *r* = 0.63	W = 66, Z = 2.93, *p* = 0.000~, *r* = 0.63	W = 66, Z = 2.93, *p* = 0.000~, *r* = 0.63	W = 66, Z = 2.93, *p* = 0.000~, *r* = 0.63	W = 66, Z = 2.93, *p* = 0.000~, *r* = 0.63	W = 66, Z = 2.93, *p* = 0.000~, *r* = 0.63	W = 65, Z = 2.85, *p* = 0.001, *r* = 0.61	W = 66, Z = 2.93, *p* = 0.000~, *r* = 0.63	W = 66, Z = 2.93, *p* = 0.000~, *r* = 0.63	W = 53, Z = 1.78, *p* = 0.042, *r* = 0.38	W = 61, Z = 2.49, *p* = 0.005, *r* = 0.53	W = 64, Z = 2.76, *p* = 0.001, *r* = 0.59
PD4	W = 45, Z = 1.07, *p* = 0.160, *r* = 0.23	W = 26, Z = −0.62, *p* = 0.740, *r* = 0.13	W = 63, Z = 2.67, *p* = 0.002, *r* = 0.57	W = 66, Z = 2.93, *p* = 0.000~, *r* = 0.63	W = 66, Z = 2.93, *p* = 0.000~, *r* = 0.63	W = 66, Z = 2.93, *p* = 0.000~, *r* = 0.63	W = 66, Z = 2.93, *p* = 0.000~, *r* = 0.63	W = 66, Z = 2.93, *p* = 0.000~, *r* = 0.63	W = 66, Z = 2.93, *p* = 0.000~, *r* = 0.63	W = 66, Z = 2.93, *p* = 0.000~, *r* = 0.63	W = 66, Z = 2.93, *p* = 0.000~, *r* = 0.63	W = 62, Z = 2.58, *p* = 0.003, *r* = 0.55	W = 56, Z = 2.04, *p* = 0.021, *r* = 0.44
PD5	W = 43, Z = 2.43, *p* = 0.006, *r* = 0.57	W = 40, Z = 2.07, *p* = 0.020, *r* = 0.49	W = 44, Z = 2.55, *p* = 0.004, *r* = 0.60	W = 45, Z = 2.67, *p* = 0.002, *r* = 0.63	W = 43, Z = 2.43, *p* = 0.006, *r* = 0.57	W = 45, Z = 2.67, *p* = 0.002, *r* = 0.63	W = 26, Z = 0.41, *p* = 0.367, *r* = 0.10	W = 45, Z = 2.67, *p* = 0.002, *r* = 0.63	W = 45, Z = 2.67, *p* = 0.002, *r* = 0.63	W = 45, Z = 2.67, *p* = 0.002, *r* = 0.63	W = 39, Z = 1.96, *p* = 0.027, *r* = 0.46	W = 38, Z = 1.84, *p* = 0.037, *r* = 0.43	W = 34, Z = 1.36, *p* = 0.102, *r* = 0.32
PD6	W = 29, Z = 0.77, *p* = 0.248, *r* = 0.18	W = 45, Z = 2.67, *p* = 0.002, *r* = 0.63	W = 39, Z = 1.96, *p* = 0.027, *r* = 0.46	W = 45, Z = 2.67, *p* = 0.002, *r* = 0.63	W = 42, Z = 2.31, *p* = 0.010, *r* = 0.54	W = 45, Z = 2.67, *p* = 0.002, *r* = 0.63	W = 45, Z = 2.67, *p* = 0.002, *r* = 0.63	W = 45, Z = 2.67, *p* = 0.002, *r* = 0.63	W = 45, Z = 2.67, *p* = 0.002, *r* = 0.63	W = 45, Z = 2.67, *p* = 0.002, *r* = 0.63	W = 42, Z = 2.31, *p* = 0.010, *r* = 0.54	W = 37, Z = 1.72, *p* = 0.049, *r* = 0.40	W = 30, Z = 0.89, *p* = 0.213, *r* = 0.21
PD7	W = 78, Z = 2.27, *p* = 0.011, *r* = 0.45	W = 53, Z = 0.52, *p* = 0.318, *r* = 0.10	W = 86, Z = 2.83, *p* = 0.000~, *r* = 0.56	W = 91, Z = 3.18, *p* = 0.000~, *r* = 0.62	W = 91, Z = 3.18, *p* = 0.000~, *r* = 0.62	W = 90, Z = 3.11, *p* = 0.000~, *r* = 0.61	W = 91, Z = 3.18, *p* = 0.000~, *r* = 0.62	W = 88, Z = 2.97, *p* = 0.001, *r* = 0.58	W = 41, Z = −0.31, *p* = 0.632, *r* = 0.06	W = 47, Z = 0.10, *p* = 0.473, *r* = 0.02	W = 78, Z = 2.27, *p* = 0.011, *r* = 0.45	W = 79, Z = 2.34, *p* = 0.009, *r* = 0.46	W = 68, Z = 1.57, *p* = 0.064, *r* = 0.31
PD8	W = 60, Z = 1.01, *p* = 0.170, *r* = 0.20	W = 69, Z = 1.64, *p* = 0.055, *r* = 0.32	W = 84, Z = 2.69, *p* = 0.002, *r* = 0.53	W = 91, Z = 3.18, *p* = 0.000~, *r* = 0.62	W = 91, Z = 3.18, *p* = 0.000~, *r* = 0.62	W = 90, Z = 3.11, *p* = 0.000~, *r* = 0.61	W = 84, Z = 2.69, *p* = 0.002, *r* = 0.53	W = 91, Z = 3.18, *p* = 0.000~, *r* = 0.62	W = 91, Z = 3.18, *p* = 0.000~, *r* = 0.62	W = 91, Z = 3.18, *p* = 0.000~, *r* = 0.62	W = 76, Z = 2.13, *p* = 0.016, *r* = 0.42	W = 91, Z = 3.18, *p* = 0.000~, *r* = 0.62	W = 87, Z = 2.90, *p* = 0.001, *r* = 0.57

$\mathrm{r}=\frac{\mathrm{Z}}{\sqrt{2\mathrm{n}}}$, n denotes the number of trials conducted in each subject

**Fig. 4:**
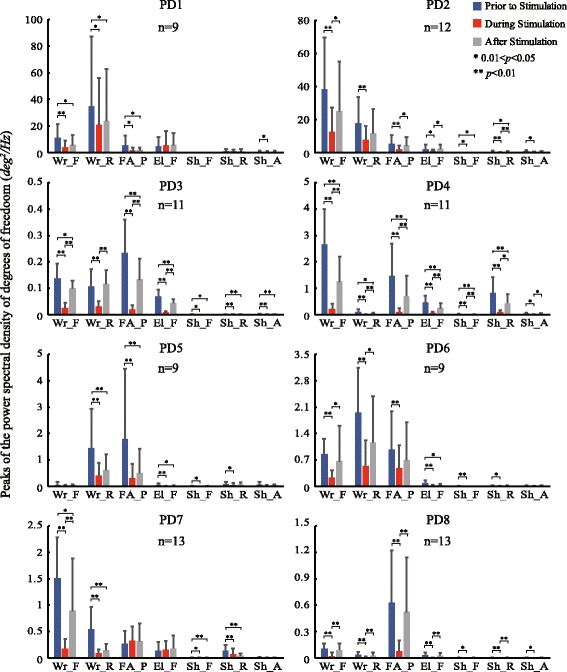
Statistical charts of tremor amplitudes of 7 DOFs in joints in 8 subjects. The tremor amplitude was the peak value between 2 to 7 Hz of the PSD. The average tremor amplitude of different trials of three time periods of 7 DOFs in joints in one subject are separately presented as bars (error bars: standard deviation). “n” denotes the total number of trials. The Wilcoxon signed-rank test was employed to show the differences of tremor amplitude between the time periods. The alternative hypothesis: P_*p*_ is greater than P_*s*_, P_*p*_ is not equal to P_*a*_, and P_*s*_ is not equal to P_*a*_. *0.01 < *p* < 0.05; **p < 0.01. P_*p*_: tremor intensity prior to stimulation; P_*s*_: tremor intensity during stimulation; P_*a*_: tremor intensity after stimulation

**Fig. 5:**
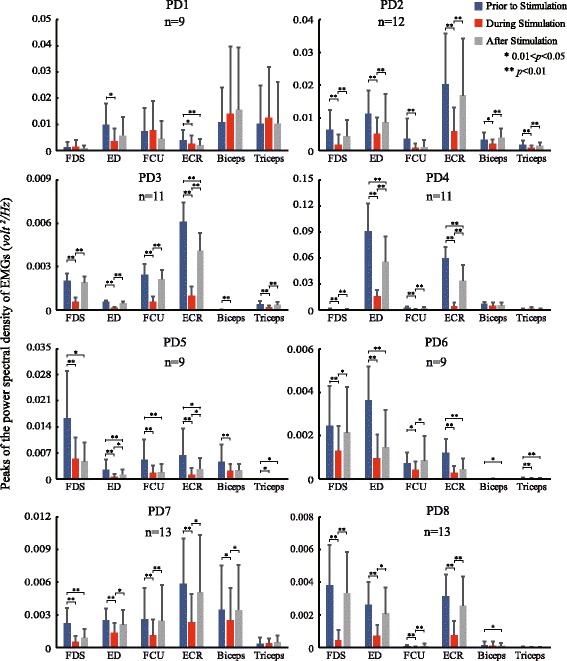
Statistical charts of tremor amplitudes of 6 EMGs in 8 subjects. The average tremor values of different trials of three time periods of 6 EMGs in one subject are separately presented as bars (error bars: standard deviation), similar as Fig. 4

Figures 4 and 5 showed the distribution of PSD peak amplitudes for the six EMGs (Fig. 5) and tremors at all DOFs (Fig. 4) prior to, during and after stimulation for all subjects. Closer examination of the changes in the PSD prior to and during stimulation in all trials for all subjects illustrated that a significant decrease in the PSD peak was apparent in most DOFs. However, in two subjects, there appeared a slight increase in the mean value of tremor during stimulation in El_F of PD1 and in FA_P and El_F of PD7 (Fig. 4). In fact, tremor augmentation during stimulation appeared in all subjects randomly, and the number of trials with tremor augmentation in each subject was reported in Table 2. Overall, in most DOFs of most subjects, tremor was inhibited significantly by cutaneous stimulation.

Significant suppression of EMGs in most muscles of most subjects was also evident during stimulation, except that augmentation in EMGs appeared mostly in FCU, Biceps and Triceps of PD1 and in Triceps of PD7 (Fig. 5), consistent with the behaviors of tremor augmentation of the two subjects. The number of trials with EMG augmentation in each subject was also reported in Table 2.

**Fig. 6:**
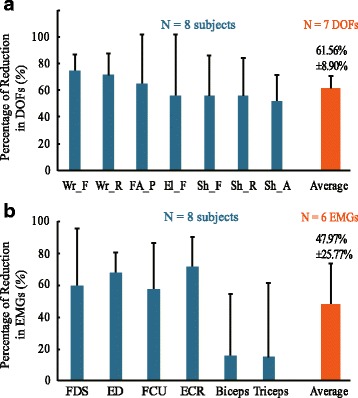
The percentage of reduction in DOFs (**a**) and EMGs (**b**) in all subjects. The individual percentage of reduction (*PR*) of each DOF (EMG) was calculated from: $PR={\frac{\sum_1^n{P}_p-\sum_1^n{P}_s}{\sum_1^n{P}_p}}^{\mathrm{x}}100\%$. The *PR* of each DOF (EMG) shown in this figure as a bar (error bar: standard deviation) was averaged from the individual *PR* across 8 subjects. The total average *PR* bars across all DOFs (EMGs) and subjects were in orange

It is clear that in most subjects, the number of augmentation in EMGs during stimulation appeared to occur randomly and much less frequently than the number of trials with suppression. Thus, the overall inhibition in EMGs and tremor of all DOFs by cutaneous stimulation was overwhelmingly evident and significant as shown in Fig. 6. The percentage of reduction in EMGs across all subjects was high for forearm muscles of FDS, ED, FCU and ECR (above 50%), but low for the Biceps and the Triceps in the upper arm (below 20%). The average percentage of reduction in EMGs across all muscles of all subjects was at 47.97%. For tremor suppression, the percentage of reduction in DOFs across all subjects was all above 50%, and the average percentage of reduction across all DOFs of all subjects was at 61.56%.

### Effects post stimulation

**Fig. 7:**
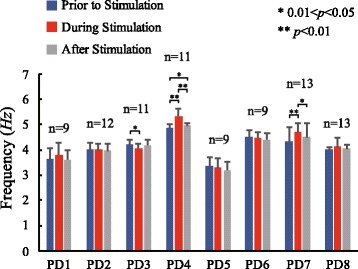
Statistical charts of tremor frequency in 8 subjects. The frequency of the peak value between 2 to 7 Hz of the PSD of each time period was denoted as tremor frequency. The average tremor frequencies of three time periods were calculated from all trials, DOFs and EMGs of one subject. The Wilcoxon signed-rank test was employed to show the differences of tremor frequency between the time periods. *0.01 < *p* < 0.05; **p < 0.01

The power spectrum densities (PSDs) of EMGs and tremor in DOFs were also estimated for the period after stimulation, and presented in Figs. 4, 5 and 7 as well. In general, tremor reemerged immediately after termination of stimulation, but with varying intensities that may be significantly different from those prior to stimulation.

Tremor frequencies in the three periods of a test were presented in Fig. 7. The statistic results showed that there was no consistent trend across subjects for any of the three comparisons. The effect of electrical stimulation on tremor frequency was not conclusive in this experiment.

## Discussion

Resting tremor remains a chronic condition in more than 70% of patients with PD. Tremor dominant patients do not respond to medication, such as Levodopa, as well as other patients with PD [1, 2, 4]. Currently, deep brain stimulation (DBS) remains the most effective medical treatment to overcome tremor, but is reserved only for patients with severe symptoms [9–11, 37]. A large population of patients with PD suffering resting tremor is without any intervention to alleviate their motor symptom. Functional electrical stimulation of muscles has been employed to counteract the trembling of limb in patients with PD [5–8]. Most of these studies attributed the effect of tremor suppression to counteractive muscle contraction. Only a few pointed out the possible effects of stimulating sensory afferents for inhibiting tremor [8]. Our results in this study demonstrate clearly that stimulating cutaneous sensory afferents can reduce tremor intensity in patients with PD.

The results of this study may be explained by the finding that electrical stimulation of cutaneous superficial radial nerve of the ipsilateral forelimb in cat could evoke strong disynaptic inhibitory postsynaptic potentials (IPSPs) [25], which were presumably via the PN network. Pierrot-Deseilligny et al. [23] summarized the evidence that the cervical propriospinal premotoneurons received strong inhibition from the cutaneous afferents in the upper limb. Cutaneous afferents were found to inhibit rigidity to a certain degree in patients with Parkinson’s disease [27, 38]. However, these studies focused either on isometric or tonic contraction of muscles, and the effects of cutaneous afferents on Parkinsonian resting tremor have not been tested yet in patients with PD. In a computational analysis, Hao et al. [24] proposed that cortical oscillations of tremor signals are processed and transmitted to peripheral muscles via the PN network. This led to the underlying hypothesis in this study that evoked cutaneous afferents TENS could affect the corticospinal transmission of tremor signals, and thus inhibiting tremor at the peripheral limb. Results here provide clear evidence to support this hypothesis as was suggested in an earlier study using computational modeling [24]. The phenomenon of tremor reduction observed in this study was also consistent with the inhibitory effects of cutaneous afferents to motor commands in humans [26, 27] and animals [23, 25]. Thus, the findings of this study were consistent with neurophysiological evidence, which corroborates the idea of inhibiting tremor by disrupting corticospinal transmission of descending tremor signals via evoked cutaneous afferents with TENS.

In this study, we observed that tremor in patients with PD is generally more severe in distal joints, such as wrist and elbow supination, but less obvious in more proximal joints, such as shoulder (Fig. 4), and so is the extent of suppression by evoked cutaneous afferents as is shown in Fig. 6(a). In an earlier analysis of EMG signals in patients with PD with tremor dominant symptom, we showed that the amplitude of tremor at joints is modulated by the number of muscles synchronized with rhythmic alternating bursts [16]. Thus, distal joints in the upper extremity are affected by a greater number of synchronized muscles, and so is tremor at distal joints. Proximal joints are acted upon by fewer synchronized muscles, therefore, experiencing less tremor. In addition, the open-chain structure of the upper extremity may also play a role in amplifying tremor at distal joints. Thus, when tremor signals are reduced by evoked cutaneous afferents, tremor at distal joints is naturally reduced by a greater extent. However, the percentage of reduction of tremor at each DOF in the upper extremity was uniform across all patients with PD (Fig. 6(a)). The percentage of reduction from distal to proximal joints ranged from 70% to 50%, with an average reduction in tremor amplitude of 61.56% (Fig. 6(a)). This result indicates that it is feasible to reduce the intensity of tremor in the upper extremity of patients with PD to a large extent with cutaneous stimulation.

One of the consistently observed behaviors in this study is gradual recovery of tremor during stimulation. This phenomenon may be associated with adaptation of skin receptors activated by electrical stimulation. Although not specific, cutaneous afferents may arise from a variety of mechanoreceptors, hair cells and even free nerve endings beneath the skin [39]. How to avoid sensory adaptation may be a relevant question in future studies. The location to place stimulation electrodes may also be important to obtain more effective inhibition. The superficial radial nerve is a relatively “pure” sensory nerve, i.e. dominated by sensory afferent fibers over motor efferent fibers. Stimulating the dorsal skin of the hand activates mostly the superficial radial nerve that gives rise to cutaneous sensory afferents. If stimulation electrodes were placed correctly to target the superficial radial nerve, a medium amplitude stimulus would cause a feeling of radiation along the index finger or the thumb [29–31]. This radiating sensation is the stimulation marker to elicit inhibiting effects (Figs. 2 and 3). It is interesting to note that a single stimulation site in the dorsal skin of hand elicited a diffused pattern of inhibition to EMGs of a set of muscles in the upper extremity. The afferent pathways of this fanning inhibition await elucidation in future studies.

Another often observed behavior is augmentation in EMGs or DOFs during cutaneous stimulation. This may be due to the multiple effective sites of cutaneous afferents by means of spinal interneuronal or transcortical loops [25–27, 40–43]. However, the random occurrence of EMG augmentation in the trials may indicate that the gains of these interneuronal or transcortical loops may be low and fluctuate. Indeed, we found in this study that at 1.5-1.75 times RT of stimulation amplitude, inhibitory effects to tremor were predominant, suggesting that a specific strength of stimulation may selectively activate afferent fibers that are overwhelmingly inhibitory to the corticospinal pathways of tremor signals.

## Conclusions

In this paper, we tested the hypothesis that tremor could be suppressed in subjects with PD by disrupting the corticospinal transmission of descending tremor signals by means of inhibitory effects of cutaneous reflex. In 8 subjects with PD recruited to participate in this study, tremor reduction in each degree of freedom was significant, and an average percentage of reduction in tremor of 61.56% was achieved. The suppression was more effective in distal joints than in proximal joints. In some patients with PD, complete suppression of tremor at the upper extremity was obtained, although augmentation in EMGs and tremor in all DOFs during cutaneous stimulation was also observed in some cases. In those patients with PD displaying partial inhibition, disinhibition of tremor may be associated with adaption of cutaneous receptors under stimulation. Results from this study supports the hypothesis that tremor can be inhibited by evoked cutaneous reflex using TENS. However, the neural mechanism of tremor inhibition awaits further elucidation. The technique may suggest a non-invasive intervention to alleviate resting tremor for a large population of patients with PD having tremor dominant symptom.

## Abbreviations

Ag/AgCl: Silver/Silver Chloride
Biceps: Biceps Brachii
DBS: Deep Brain Stimulation
DOF: Degree of Freedom
ECR: Extensor Carpi Radialis
ED: Extensor Digitorum
El_F: Elbow Flexion/Extension
EMG: Electromyography
F_*a*_: Tremor Frequency after Stimulation
FA_P: Forearm Pronation/Supination
FCU: Flexor Carpi Ulnaris
FDS: Flexor Digitorum Superficialis
F_*p*_: Tremor Frequency prior to Stimulation
F_*s*_: Tremor Frequency during Stimulation
IPSP: Inhibitory Postsynaptic Potential
MN: Motor Neuron
P_*a*_: Tremor Intensity after Stimulation
PD: Parkinson’s Disease
PN: Propriospinal Neuron
P_*p*_: Tremor Intensity prior to Stimulation
*PR*: Percentage of Reduction
P_*s*_: Tremor Intensity during Stimulation
PSD: Power Spectral Density
RT: Radiating Threshold
Sh_A: Shoulder Adduction/Abduction
Sh_F: Shoulder Flexion/Extension
Sh_R: Humeral Rotation
TENS: Transcutaneous Electrical Nerve Stimulation
Triceps: Triceps Brachii
Wr_F: Wrist Flexion/Extension
Wr_R: Wrist Radial/Ulnar Flexion
W-test: Wilcoxon Signed-rank Test

## References

[1] Hughes AJ, Daniel SE, Blankson S, Lees AJ (1993). A clinicopathologic study of 100 cases of Parkinson’s disease. Arch Neurol.

[2] Jankovic J (2008). Parkinson’s disease: clinical features and diagnosis. J Neurol Neurosurg Psychiatry.

[3] Hariz G-M, Forsgren L (2011). Activities of daily living and quality of life in persons with newly diagnosed Parkinson’s disease according to subtype of disease, and in comparison to healthy controls: ADL in early Parkinson’s disease. Acta Neurol Scand.

[4] Jankovic J, Aguilar LG (2008). Current approaches to the treatment of Parkinson’s disease. Neuropsychiatr Dis Treat.

[5] Javidan M, Elek J, Prochazka A (1992). Attenuation of pathological tremors by functional electrical stimulation. II: Clinical evaluation. Ann Biomed Eng.

[6] Popović Maneski L, Jorgovanović N, Ilić V, Došen S, Keller T, Popović MB (2011). Electrical stimulation for the suppression of pathological tremor. Med Biol Eng Comput.

[7] Prochazka A, Elek J, Javidan M (1992). Attenuation of pathological tremors by functional electrical stimulation. I: Method. Ann Biomed Eng.

[8] Dosen S, Muceli S, Dideriksen JL, Romero JP, Rocon E, Pons J (2015). Online tremor suppression using electromyography and low-level electrical stimulation. IEEE trans. Neural Syst Rehabil Eng Publ IEEE Eng Med Biol Soc.

[9] Kumar R, Lozano AM, Sime E, Lang AE (2003). Long-term follow-up of thalamic deep brain stimulation for essential and parkinsonian tremor. Neurology.

[10] Benabid AL, Chabardes S, Mitrofanis J, Pollak P (2009). Deep brain stimulation of the subthalamic nucleus for the treatment of Parkinson’s disease. Lancet Neurol.

[11] Heida T, Wentink EC, Marani E (2013). Power spectral density analysis of physiological, rest and action tremor in Parkinson’s disease patients treated with deep brain stimulation. J. Neuroengineering Rehabil..

[12] Deuschl G, Raethjen J, Baron R, Lindemann M, Wilms H, Krack P (2000). The pathophysiology of parkinsonian tremor: a review. J Neurol.

[13] Hallett M (2014). Tremor: pathophysiology. Parkinsonism Relat Disord.

[14] Helmich RC, Hallett M, Deuschl G, Toni I, Bloem BR (2012). Cerebral causes and consequences of parkinsonian resting tremor: a tale of two circuits?. Brain J Neurol.

[15] Timmermann L, Gross J, Dirks M, Volkmann J, Freund H-J, Schnitzler A (2003). The cerebral oscillatory network of parkinsonian resting tremor. Brain J Neurol.

[16] He X, Hao M-Z, Wei M, Xiao Q, Lan N (2015). Contribution of inter-muscular synchronization in the modulation of tremor intensity in Parkinson’s disease. J Neuroengineering Rehabil.

[17] Timmermann L, Fink GR (2011). Pathological network activity in Parkinson’s disease: from neural activity and connectivity to causality?. Brain J Neurol.

[18] Alstermark B, Isa T, Pettersson L-G, Sasaki S (2007). The C3-C4 propriospinal system in the cat and monkey: a spinal pre-motoneuronal centre for voluntary motor control. Acta Physiol Oxf Engl.

[19] Pierrot-Deseilligny E, Burke D. The circuitry of the human spinal cord: its role in motor control and movement disorders. New York: Cambridge University Press; 2005.

[20] Burke D, Gracies JM, Mazevet D, Meunier S, Pierrot-Deseilligny E (1994). Non-monosynaptic transmission of the cortical command for voluntary movement in man. J Physiol.

[21] Mazevet D, Pierrot-Deseilligny E (1994). Pattern of descending excitation of presumed propriospinal neurones at the onset of voluntary movement in humans. Acta Physiol Scand.

[22] Nielsen J, Pierrot-Deseilligny E (1991). Pattern of cutaneous inhibition of the propriospinal-like excitation to human upper limb motoneurones. J Physiol.

[23] Pierrot-Deseilligny E (1996). Transmission of the cortical command for human voluntary movement through cervical propriospinal premotoneurons. Prog Neurobiol.

[24] Hao M, He X, Xiao Q, Alstermark B, Lan N (2013). Corticomuscular transmission of tremor signals by propriospinal neurons in Parkinson’s disease. PLoS One.

[25] Alstermark B, Lundberg A, Sasaki S (1984). Integration in descending motor pathways controlling the forelimb in the cat. 11. Inhibitory pathways from higher motor centres and forelimb afferents to C3-C4 propriospinal neurones. Exp Brain Res.

[26] Fuhr P, Friedli WG (1987). Electrocutaneous reflexes in upper limbs--reliability and normal values in adults. Eur Neurol.

[27] Fuhr P, Zeffiro T, Hallett M (1992). Cutaneous reflexes in Parkinson’s disease. Muscle Nerve.

[28] Hao M-Z, He X, Kipke DR, Lan N. Effects of electrical stimulation of cutaneous afferents on corticospinal transmission of tremor signals in patients with Parkinson’s disease. 2013 6th International IEEE/EMBS Conference on Neural Engineering (NER). 2013. p. 355–8.

[29] Walsh DM, Foster NE, Baxter GD, Allen JM (1995). Transcutaneous electrical nerve stimulation. Relevance of stimulation parameters to neurophysiological and hypoalgesic effects. Am J Phys Med Rehabil Assoc Acad Physiatr.

[30] Zehr EP, Chua R (2000). Modulation of human cutaneous reflexes during rhythmic cyclical arm movement. Exp Brain Res.

[31] Zehr EP, Kido A (2001). Neural control of rhythmic, cyclical human arm movement: task dependency, nerve specificity and phase modulation of cutaneous reflexes. J Physiol.

[32] Pierrot-Deseilligny E (2002). Propriospinal transmission of part of the corticospinal excitation in humans. Muscle Nerve.

[33] Raethjen J, Austermann K, Witt K, Zeuner KE, Papengut F, Deuschl G (2008). Provocation of Parkinsonian tremor. Mov. Disord. Off. J. Mov. Disord. Soc.

[34] Welch P (1967). The use of fast Fourier transform for the estimation of power spectra: a method based on time averaging over short, modified periodograms. IEEE Trans Audio Electroacoustics.

[35] Woolson RF. Wilcoxon Signed-Rank Test. In: D’Agostino RB, Sullivan L, Massaro J, editors. Wiley Encycl. Clin. Trials. Hoboken, NJ: Wiley, Inc.; 2008. http://doi.wiley.com/10.1002/9780471462422.eoct979. Accessed 12 Feb 2016.

[36] R Core Team. R: A Language and Environment for Statistical Computing. Vienna: R Foundation for Statistical Computing; 2017. https://www.r-project.org. Accessed 11 Jan 2016.

[37] Johnson MD, Miocinovic S, McIntyre CC, Vitek JL (2008). Mechanisms and targets of deep brain stimulation in movement disorders. Neurother J Am Soc Exp Neurother.

[38] Pol S, Vidailhet M, Meunier S, Mazevet D, Agid Y, Pierrot-Deseilligny E (1998). Overactivity of cervical premotor neurons in Parkinson’s disease. J Neurol Neurosurg Psychiatry.

[39] Abraira VE, Ginty DD (2013). The sensory neurons of touch. Neuron.

[40] Chen R, Ashby P (1993). Reflex responses in upper limb muscles to cutaneous stimuli. Can J Neurol Sci J Can Sci Neurol.

[41] Clouston PD, Kiers L, Menkes D, Sander H, Chiappa K, Cros D (1995). Modulation of motor activity by cutaneous input: inhibition of the magnetic motor evoked potential by digital electrical stimulation. Electroencephalogr Clin Neurophysiol.

[42] Cavallari P, Fournier E, Katz R, Malmgren K, Pierrot-Deseilligny E, Shindo M (1985). Cutaneous facilitation of transmission in Ib reflex pathways in the human upper limb. Exp Brain Res.

[43] Evans AL, Harrison LM, Stephens JA (1989). Task-dependent changes in cutaneous reflexes recorded from various muscles controlling finger movement in man. J Physiol.

